# Adherence to antiretroviral therapy and viral suppression: Analysis of three periods between 2011 and 2017 at an HIV-AIDS center, Brazil

**DOI:** 10.3389/fphar.2023.1122018

**Published:** 2023-03-31

**Authors:** Micheline Marie Milward de Azevedo Meiners, Igor Araújo Cruz, Maria Inês de Toledo

**Affiliations:** ^1^ Programa de Pós-Graduação em Medicina Tropical, Faculdade de Medicina, Universidade de Brasília, Brasília, Brazil; ^2^ Curso de Farmácia e Grupo de Pesquisa Acesso a Medicamentos e Uso Responsável (AMUR), Faculdade de Ceilândia, Universidade de Brasília, Brasília, Brazil

**Keywords:** HIV, medication adherence, anti-HIV agents, viral load, viral suppression

## Abstract

The increased effectiveness of antiretroviral therapy (ART) in the last 30 years is a scientific landmark, and viral suppression is directly associated with treatment adherence. The aim of this study was to compare the results of ART adherence and viral load suppression with the evolution of the protocols and other associated factors, in people living with HIV. A panel analysis of three descriptive longitudinal studies investigating ART adherence and viral load suppression was conducted in people with HIV treated at a drug dispensing unit in the Federal District. The studies were carried out during periods of 2011, 2013, and 2017, coinciding with the three different recommended treatment schemes for the country. Adherence was assessed using drug dispensing records. Viral load data were obtained from the Ministry of Healthʼs Laboratory Examination Information System. Analysis of the data of 522 individuals in the three periods showed sociodemographic differences such as a decline in the percentage of women (from 33% in period 1 to 4% in period 3) and an increase in the percentage of young people. ART adherence was higher in period 2 (tenofovir/lamivudine/efavirenz scheme). Viral load suppression was greater in period 3 (tenofovir/lamivudine/dolutegravir scheme). The relative detectable viral load risk was nearly two-fold higher (RR 1.83) in people living with HIV with less than 80% adherence when compared to those above 80%. With respect to the different schemes recommended in Brazil during the periods studied, ART containing dolutegravir was the most effective in achieving viral load suppression. By contrast, there was better ART adherence in the daily combined fixed dose consisting of tenofovir/lamivudine/efavirenz in tablet form. Adherence to ART above 80% seemed to be enough to promote an effective treatment in therapeutic schemes including efavirenz or dolutegravir.

## Introduction

Since 1996, Brazilian Ministry of Health Law 9.313/96 (Brasil, 1996) has guaranteed free access to ART to treat HIV/AIDS, thereby lowering mortality and morbidity and contributing to improving the quality of life of people living with HIV (PLHIV) (Lima et al., 2012).

On average, 40,000 new HIV/AIDS cases have been recorded every year since 2011. In the Federal District (DF), 10,735 cases (7,903 men and 2,832 women) were reported between the first notification of the disease in 1985 and December 2017. In 2017 alone 645 new cases were recorded, a significant increase compared to previous years (SES-DF, 2018; MS, 2021).

The increase in ART effectiveness against HIV infection in the last 30 years is a landmark for global science, transforming a highly lethal acute disease into a chronic condition, with a reduction in viral transmission both congenitally and through sexual contact, over a short time period. The success of ART in suppressing viral replication, however, is directly associated with patient adherence to treatment (Trickey et al., 2017; Melo et al., 2018).

Treatment adherence is a dynamic multifactorial process involving the behavior of PLHIV, according to the treatment indicated by the health professional, using the drugs at specified doses and times, undergoing laboratory tests and periodically accessing specialized health services (da Silva et al., 2022). The extrinsic and intrinsic factors of adherence are adverse drug reactions, professional attention in guiding the therapeutic plan, access to drug dispensing units (DDU) (extrinsic) and socioeconomic conditions, psychological status, physical or cognitive limitations (intrinsic) (da Silva et al., 2022).

Studies demonstrate that there is no statistically significant difference in the likelihood of virological failure between PLHIV who achieve 95%–100% adherence and those with 80%–95%, suggesting that even the latter can suppress viral replication and not develop ART resistance (Bezabhe et al., 2016; Miyada et al., 2017).

The aim of the present study was to compare treatment adherence and viral load (VL) suppression with the evolution of the protocols and other associated factors in PLHIV who received ART at a DDU in the DF and participated in longitudinal studies conducted in three different guideline periods for the care of adults infected by HIV.

## Materials and methods

### Study design, location and population

This is a panel analysis of three descriptive longitudinal studies carried out at different times at the Teaching Pharmacy of the Hospital Universitário de Brasilia (TP-HUB), a DDU specialized in treating PLHIV in the DF (Cunha, 2014; Fagundes, 2017; Batista, 2018).

The three databases used in the study sample were classified according to the recommendation or protocol in effect at the time of the research (Table 1).

**TABLE 1:** Database characteristics in each study period. Hospital Universitário de Brasilia, Brasilia, Brazil, 2019.

Study period	Period 1	Period 2	Period 3
	RTAAHIV^a^ (2008)	PCDT^b^ (2013)	PCDT^b^ (2017)
Treatment phase	Initial and experiments	Only at the start of treatment	Only at the start of treatment
Initial ART	Several therapeutic schemes	TDF/3TC/EFZ	TDF/3TC + DTG
Observation time	January 2011 to June 2013 (30 months)	January 2014 to December 2016 (36 months)	May to September 2017 (12 months)

^a^ Recommendations for Antiretroviral Therapy in Adults Infected with HIV.

^b^ Clinical Protocol and Therapeutic Guidelines (PCDT) for Managing HIV, infection in adults; ART, Antiretroviral Therapy.

It is important to describe the characteristics of each treatment for adults with HIV/AIDS at the different times in Brazil:

- Period 1: August 2008 to September 2013, the Recommendations for Antiretroviral Therapy in Adults Infected with HIV stipulated that to initiate treatment, individuals had to exhibit symptoms of at least one of the diseases that characterize AIDS and/or have a CD4^+^ count < 200 cel/mm³, but treatment for asymptomatic cases and CD4^+^ between 350 and 200 cel/mm³ could also be considered. The first-line drugs were zidovudine, lamivudine and efavirenz (AZT/3TC + EFZ), dispensed in separate “2 in 1” + 1 tablets (MS, 2008).
- Period 2: October 2013 to March 2017, the Clinical Protocol and Therapeutic Guidelines (PCDT) for Managing HIV Infection in Adults of 2013 established ART for people with CD4^+^ less than or equal to 500 cel/mm³ and in cases of coinfection with hepatitis B (HBV), the presence of neoplasia or high-risk cardiovascular diseases, but already recommended the early application of ART (MS, 2013). First-line drugs were TDF/3TC/EFZ administered in a fixed dose combined in a single tablet.
- Period 3: finally, from April 2017 to the present PCDT-2017 established ART for PLHIV after diagnosis, irrespective of VL and CD4^+^ levels or other clinical conditions based on evidence of treatment as prevention (TasP) (MS, 2017).

The common inclusion criteria in the three studies were PLHIV aged 18 years or older, treated at the HUB outpatient clinic and who underwent ART at TP-HUB. The exclusion criteria in the periods studied were pregnant women, <18 years old, people who used ART as prophylaxis, PLHIV who died before 60 days of treatment, stopped taking medication before 60 days of treatment, had initiated treatment less than 60 days before, whose medical records were not found and duplicate records. In the period 2 and 3 studies, patients at the onset of treatment were selected. Period 1 included PLHIV undergoing treatment initiated at any time. Participants are exclusive to each study and do not take part in the others.

### Information sources and data analysis

In order to measure adherence, all the studies used drug dispensing records obtained from the Logistics Drug Control System (SICLOM) in the operational mode. This information system is part of the Ministry of Health’s strategies for managing HIV/AIDS in the country, in conjunction with the Laboratory Examination Control System (SISCEL), where viral load (VL) data were obtained.

The calculation and classification of the dependent variable ART adherence, for periods 1, 2, and 3, were redone for standardization purposes. Adherence was calculated using the pharmacy dispensing records, which have been used by several authors to assess adherence over 12 months or 12 dispensations (da Costa Lima-Dellamora et al., 2017; Yager et al., 2017).

Based on these data, adherence was classified, according to the literature, as optimal (dispensation record greater than or equal to 95% of the period analyzed), good (between 80 and less than 95%) and low (less than 80%) (Castillo-Mancilla and Haberer, 2018; Dunn et al., 2018). For purposes of the statistical analysis of relative risk, adherence was classified as optimal/good (dispensation record greater than or equal to 80% of the period analyzed) and low (less than 80%).

The dependent variable final VL was extracted from the SISCEL after 6 months (± 30 days) of treatment and stratified as not detectable (ND) for those with fewer than 50; 50 to 1,000; 1,000 to 100,000; and >100,000 copies/mL. For purposes of the statistical analysis of relative risk, the final VL was dichotomized and categorized as ND (<50 copies/mL) or detectable (≥ 50 copies/mL).

The common independent variables in the three periods were sex, age (in years during each period), initial ART therapeutic scheme, treatment time (days), and number of treatment changes (zero, once, twice or more). Initially, all the database variables were analyzed by descriptive statistics (mean and standard deviation; relative and absolute frequency). The continuous variables were subsequently categorized into ranges and categorical variables described as frequencies and percentages.

For the continuous independent variable initial VL, the same stratification as final VL was used. There were no diagnostic data records for 28% of PLHIV, on either medical charts or the SISCEL database.

After normal distribution asymmetry and homogeneity in only two variables (age and final VL) was determined in each database (Shapiro-Wilk and Levene tests, respectively), Pearson’s chi-squared and Fisher’s Exact tests were conducted to analyze factors associated with the variables of interest. Analyses included relative risk and its respective confidence intervals (95% CI). The Mann-Whitney test was applied to identify the correlated variables. *p*-values less than 5% (*p* < 0.05) were considered significant. The databases were structured in the Microsoft Excel^®^ program. Statistical analyses were carried out using the SPSS^®^ program (Statistical Package for the Social Sciences), IBM, version 20.0.

## Results

**FIGURE 1:**
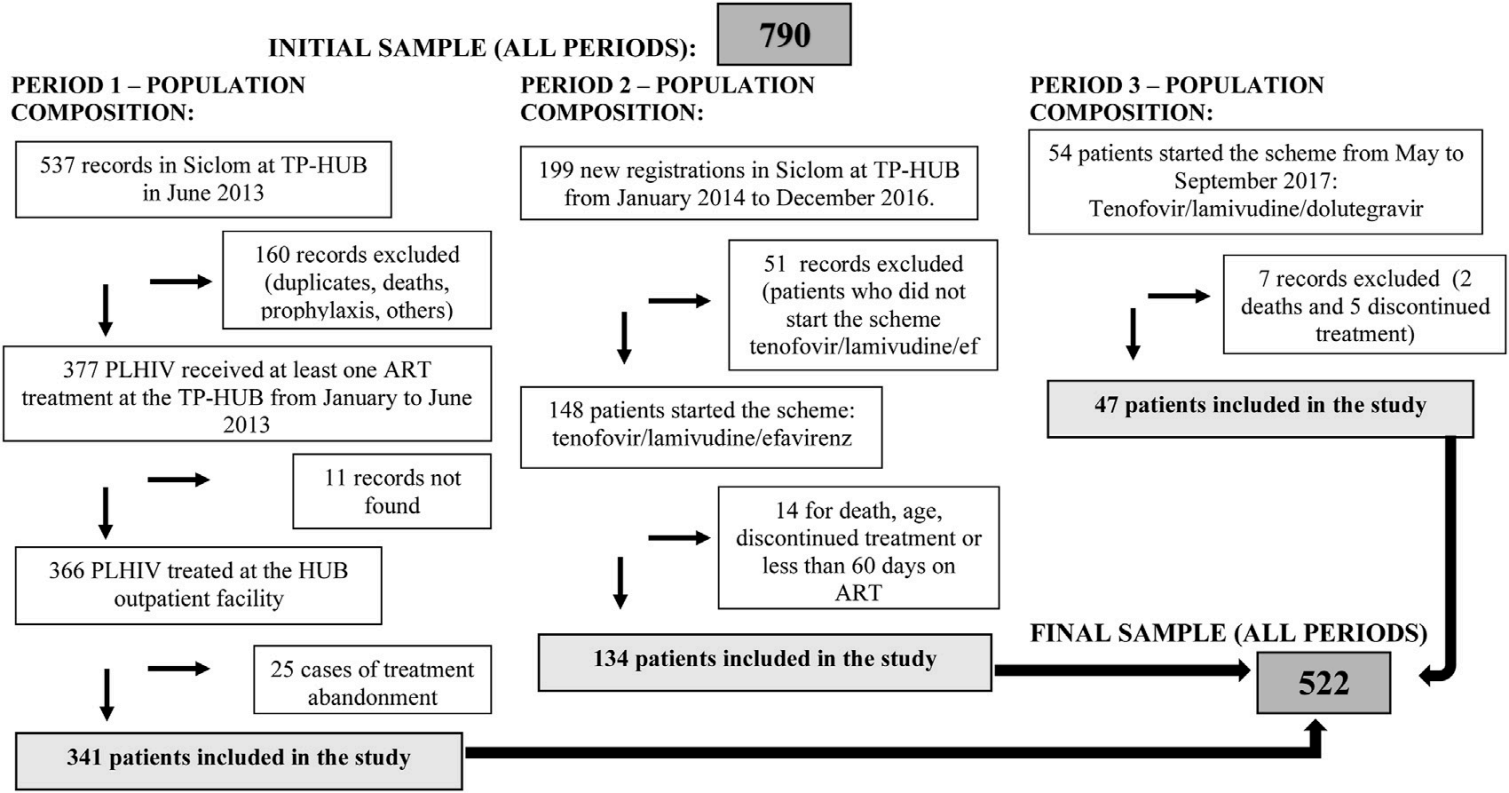
Flowchart with the composition of the sample in each study period. Hospital Universitário de Brasilia, Brasilia, Brazil, 2019.

The composition of the three databases used in the study is presented in Figure 1. The databases contained 790 PLHIV. The inclusion and exclusion criteria resulted in 268 people being excluded for the different reasons described. The final sample size was 522 PLHIV.

The 522 PLHIV included a majority (75.1%) of men, with an average age of 40.6 years (standard deviation of 10.8 years), as shown in Table 2. In period 1, only 129 individuals (37.8%) had preferential initial ART (AZT+3TC + EFZ), according to the recommendation used at the time (2008), while for periods 2 and 3, it was an inclusion criterion, that is, 100% of patients underwent preferential initial ART according to the respective protocol. ART was modified in 102 PLHIV during the three periods, with a total of 126 alterations (Table 2). The average percentage of ART adherence across all three periods was 90.7%, with PLHIV in period 2 exhibiting the best adherence (92.4%) (Table 2).

**TABLE 2:** Sociodemographic and therapeutic characteristics of people living with HIV/AIDS in the study periods (*n* = 522), Hospital Universitário de Brasilia, Brasilia, Brazil, 2019.

Variables	Period 1 (2011–2013)^a^ *n* = 341	Period 2 (2014–2016)^a^ *n* = 134	Period 3 (2017-2018)^a^ *n* = 47	Total *N* = 522
Sex (%)				
Men	229 (67.2)	118 (88.1)	45 (95.7)	392 (75.1)
Women	112 (32.8)	16 (11.9)	2 (4.3)	130 (24.9)
Age Range (%)^b^				
18 to 29	28 (8.2)	59 (44.0)	16 (34.0)	103 (19.7)
30 to 39	94 (27.6)	42 (31.3)	21 (44.8)	157 (30.1)
40 to 49	127 (37.2)	23 (17.2)	8 (17.0)	158 (30.3)
50 to 59	71 (20.8)	8 (6.0)	1 (2.1)	80 (15.3)
≥ 60	21 (6.2)	2 (1.5)	1 (2.1)	24 (4.6)
Age Mean (SD)^c^	44.2 (9.89)	33.8 (9.55)	33.9 (8.05)	40.6 (10.8)
Treatment Time, days, Mean (SD)^c^	588 (179.37)	377 (163.07)	346 (57.05)	512.6 (197.6)
PLHIV^d^ with changes to the ART scheme (%)^e^				
0	269 (78.9)	106 (79.1)	45 (95.8)	420 (80.5)
1	59 (17.3)	23 (17.2)	1 (2.1)	83 (15.9)
2 or more	13 (3.8)	5 (3.7)	1 (2.1)	19 (3.6)
Percentage adherence^f^, Mean (SD)^c^	90.4 (14.71)	92.4 (12.43)	87.6 (21.0)	90.7 (14.9)

Source: Antiviral dispensing data and sociodemographic information were extracted from the Logistics Drug Control System (SICLOM/MOH).

^a^ Different treatment guidelines were in force during the study periods: Period 1 - Recommendations for Antiretroviral Therapy in Adults Infected with HIV (2007); Period 2 - Clinical Protocol and Therapeutic Guidelines (PCDT) for Managing HIV, Infection in Adults (2013), Period 3 - Clinical Protocol and Therapeutic Guidelines (PCDT) for Managing HIV, Infection in Adults (2017).

^b^ Age during each stage of the study.

^c^ SD: standard deviation.

^d^ PLHIV; people living with HIV/AIDS.

^e^ ART: antiretroviral therapy.

^f^ Adherence percentage calculated from the ART, dispensing records of the pharmacy.

The highest percentage of VL suppression (88.9%) occurred in period 3 (Table 3). This variables had an important missing data, with participants with no viral load examination records in the system. There were 28% of subjects without information for initial VL and 33.5% for final VL.

**TABLE 3:** Initial and final viral load range of people living with HIV included in the study for the periods assessed, Hospital Universitário de Brasilia, Brasilia, Brazil, 2019.

Variables	ND	50–1,000	1,000–100,000	> 100,000
Period 1	*n* (%)	*n* (%)	*n* (%)	*n* (%)
VLs^a^ (*N* = 269)	58 (21.6)	35 (13.0)	112 (41.6)	64 (23.8)
VL_f_ ^b^ (*N* = 241)	192 (79.7)	24 (10.0)	20 (8.3)	5 (2.1)
Period 2				
VLs^a^ (*N* = 79)	3 (3.8)	11 (13.9)	42 (53.2)	23 (29.1)
VL_f_ ^b^ (*N* = 79)	57 (72.2)	14 (17.7)	7 (8.9)	1 (1.3)
Period 3				
VLs^a^ (*N* = 28)	7 (25.0)	4 (14.3)	11 (39.3)	6 (21.4)
VL_f_ ^b^ (*N* = 27)	24 (88.9)	-	1 (3.7)	2 (7.4)
Total				
VLs^a^ (*N* = 376)	68 (18.1)	50 (13.3)	165 (43.9)	93 (24.7)
VL_f_ ^b^ (*N* = 347)	273 (78.7)	38 (11.0)	28 (8.1)	8 (2.3)

Source: Viral load data of people living with HIV, were extracted from the Laboratory Examination Control System (SISCEL/MOH).

^a^ VL_s_: Viral load at the start of the treatment

^b^ VR_f_, Viral load at the end of the study.

^c^ Not detectable. Observation: participants with no viral load examination records in the system: VLs, Period 1 = 72, Period 2 = 55, Period 3 = 19, Total = 146 (28%); VLf, Period 1 = 100, Period 2 = 55, Period 3 = 20, Total = 175 (33.5%).

Analysis of adherence in relation to the independent variables showed a significant difference when changes in antiretroviral therapy occurred (*p* = 0.031). There was greater adherence in women, but lower for those aged between 18 and 29 and 40–49 years (Table 4).

**TABLE 4:** Antiretroviral therapy adherence in people living with HIV/AIDS, according to demographic, therapeutic and clinical characteristics, Hospital Universitário de Brasilia, Brasilia, Brazil, 2019.

Variables	Optimal Adherence^a^%	Good adherence^a^%	Low Adherence^a^%	*p*-value
Sex (*n* = 522)				0.606
Male	60.5	20.0	17.7	
Female	62.3	24.0	15.6	
Age Range (years, *n* = 522)				0.374
18–29	61.2	19.4	19.4	
30–39	65.0	21.7	13.4	
40–49	55.7	24.7	19.6	
Older than 50	62.5	26.0	11.5	
Change in ART (*n* = 522)				0.031
Yes	52.9	22.5	24.6	
No	62.9	23.1	14.0	
Initial viral load (*n* = 371)				0.267
ND^b^	67.6	19.1	13.2	
50–1,000	54.0	26.0	20.0	
1,000- 100,000	62.4	24.8	12.7	
> 100,000	55.9	20.4	23.7	
Total	60.9	23.0	16.1	

^a^ Adherence classification: Optimal ≥ 95%; Good, between > 80% and < 95%, and Low ≤ 80%.

^b^ ND: not detectable, fewer than 50 copies.

In relation to final VL (Table 5), the lower VL suppression was significant in patients who changed their ART (*p*= <0.001| RR 1.32; 95%CI 1.07–1.63) and higher in those with initial VR values less than 100,000 copies/mL (*p* = 0.01) and individuals with optimal (>95%) and good (80%–95%) treatment adherence (*p*= <0.001). It is important to note that PLHIV with low adherence (<80%) exhibited a nearly two-fold higher risk of not suppressing VL (RR 1.83; 95% CI 1.37–2.34) compared to those with optimal and good adherence (above 80%). The 18-29-year age group exhibited a higher percentage of detectable final VL (Table 5) than that of the other age groups, albeit not statistically significant.

**TABLE 5:** Viral load in people living with HIV/AIDS, according to demographic, therapeutic, initial viral load and adherence, Hospital Universitário de Brasilia, Brasilia, Brazil, 2019.

Variables	% VL detectable^a^	% VL not detectable	*p*-value	RR^b^ (95%CI)
Sex (*n* = 347)			0.345	
Male	20.2	79.8		
Female	25.0	75.0		
Age (years, *n* = 347)			0.148	
18–29	29.2	70.8		
30–39	22.4	77.6		
40–49	21.1	78.9		
Older than 50 years	13.3	86.7		
Change in ART^c^ (*n* = 341)			<0.001	1.32 (1.07–1.63)
Yes	37.9	62.1		
No	18.0	82.0		
Initial viral load (*n* = 328)			<0.001	
ND	5.7	94.3		
50–1,000	17.8	82.2		
1,000–100,000	20.3	79.7		
>100,000	35.4	64.6		
Adherence^d^ (*n* = 347)			<0.001	1.83 (1.37–2.43)
Low	53.6	46.4		
Optimal/good	15.1	84.9		
Total	21.3	78.7		

^a^ VL: Viral Load.

^b^ RR: Relative Risk.

^c^ ART: Antiretroviral therapy.

^d^ Adherence classificaion: Optimal/good > 80%, and Low ≤80%.

## Discussion

This study analyzed the treatment adherence and VL suppression of PLHIV using ART treatment at a DDU, based on the data from three studies that occurred at the same service but at different times, describing the evolution of guidelines for the care of adults with HIV in Brazil (Cunha, 2014; Fagundes, 2017; Batista, 2018). No other literature studies were found that compared and analyzed ART adherence and viral suppression at three different periods, when recommendations or clinical protocols for HIV/AIDS were implemented in the country.

The study showed that PLHIV with low adherence exhibited a two-fold higher risk of not suppressing VL with the use of ART than those with optimal or good adherence. In our findings, adherence above 80% achieved viral suppression in around 90% of the population, results compatible with a previous meta-analysis (Pacheco et al., 2019). The goal proposed by UNAIDS, denominated 90–90–90 (90% of infected people should be diagnosed, 90% should be in treatment and 90% should reach viral suppression), is feasible if countries achieve good adherence rates (Nachega et al., 2016).

A predominance of men was found in the PLHIV studies, in all the periods, with a marked decline in women in periods 2 and 3. The decrease in new cases among women has been reported in national epidemiological data, with an increase in the men: women ratio to 2.3 in 2018 (MS, 2021), and also described in the international literature (WHO, 2019).

With respect to age group, there was a change in HIV incidence in younger people. In period 1, around 40% of PLHIV were aged between 18 and 39 years, while in periods 2 and 3 the percentage doubled (around 75% and 80%, respectively). This change in the profile of new PLHIV is attracting attention in HIV/AIDS treatment adherence studies, especially in adolescents and young people in peripheral areas, due to the risks that these age groups are exposed to, such as social vulnerability, injectable drug sharing, and unprotected sex, among others (Dunn et al., 2018; Taquette and Rodrigues, 2019). In addition, this population exhibited a concerning higher viral load at the end of the study period. Specific care strategies are needed for this population in order to reduce the impact of HIV/AIDS in the coming decades (MS, 2019; WHO, 2019).

Over time, there has been a change in the clinical profile of PLHIV who use ART in Brazil, according to each new guideline adopted by the Ministry of Health. In terms of therapeutic schemes, in periods 2 and 3, the preferential scheme was the inclusion criteria, as determined by PCDT-2013 and its update in 2017. In period 1, in addition to PLHIV in previous treatment, the 2008 recommendation did not mandate ART as the recommended scheme or justify ART changes on the Drug Request Form, as occurred after PCDT-2013, which explains the low percentage of the preferential scheme during this period (Pavlos and Phillips, 2011).

There was better ART adherence in period 2 (92.4%) than in the other periods. Our results demonstrated that introducing a combined fixed dose, such as tenofovir, lamivudine and efavirenz (TDF/3TC/EFZ), administered once a day, improved adherence. As reported in the literature, simpler dosing schemes, with fewer administrations and unique dosage forms favor adherence (Bisson et al., 2008; Cotte et al., 2017; Costa et al., 2018; Altice et al., 2019). Furthermore, intrinsic and extrinsic factors may affect treatment adherence (Toledo et al., 2019).

Viral load suppression was higher in period 3 (88.9%), despite its lower adherence rate in relation to the other periods. One of the main changes in PCDT-2017 was replacing EFZ with dolutegravir (DTG) in the preferential scheme for initial treatment of PLHIV or in cases of people with EFZ-related adverse reactions (MS, 2018; Benzaken et al., 2019; MS, 2021). The results suggest that the scheme containing DTG is more effective than the earlier first-line schemes (Batista et al., 2019). International studies and guidelines suggest that the use of DTG in ART achieved good viral load suppression in six months, with a low percentage of adverse reactions and scheme changes (Corado et al., 2018). However, other studies have shown an increasing number of adverse events attributed to DTG (neuropsychiatric symptoms, weight gain and systemic hypersensitivity reactions) (Scévola et al., 2020).

Also significant was the relation between viral suppression for PLHIV and initial VL up to 100,000 copies and treatment adherence. PCDT-2017 recommended that ART be prescribed as soon as HIV is diagnosed. This recommendation was based on evidence that viral suppression is faster with early treatment (Bigna et al., 2016). The “non-transmissible” concept proposed by the Ministry of Health (MS, 2019), can be used for PLHIV in treatment exhibiting undetectable VL examinations for more than six months, according to the literature, used as the basis for this new concept (Rodger et al., 2019). This information reinforces the importance of ART adherence as a means of preventing HIV, thereby avoiding transmission of the virus and gradually reducing the budgetary impact on the health system (Pacheco et al., 2019).

Study limitations include the fact that around 30% of participating PLHIV had no VL registered in SISCEL, after the onset of ART, which may have produced biased results. Some of the individuals may have undergone examinations in private laboratories and these data were not included in SISCEL, which may compromise future research on strategic public health measures for PLHIV (Benzaken et al., 2019; Focaccia et al., 2019; Meireles et al., 2019). Adherence was measured using the pharmacy dispensing register, which, despite being used in other Brazilian studies, exhibits limitations (Gachara et al., 2017). Moreover, the Medical records contain incomplete sociodemographic and clinical information, even for the most recent period, thereby hindering analyses of other independent variables.

## Final considerations

Adherence is an important factor in achieving effective treatment for chronic conditions, such as HIV/AIDS. Simplified therapeutic schemes consisting of a combined fixed single tablet dose, such as in the TDF/3TC/EFZ scheme used in the second period of the study, favor treatment adherence. However, despite the lower adherence (>80%), treatments containing dolutegravir seem to promote and maintain viral suppression in around 90% of PLHIV. Based on these results, adherence above 80% achieves effective viral suppression in therapeutic schemes involving efavirenz and dolutegravir.

## Data Availability

The raw data supporting the conclusion of this article will be made available by the authors, without undue reservation.
